# Integrated reconstruction of January and July temperature series in the Sanjiangyuan region during the Holocene

**DOI:** 10.1371/journal.pone.0337521

**Published:** 2026-01-20

**Authors:** Yan Wang, Guangchao Cao, Guangliang Hou, Jian Ni, Tao Huang, Li Yan, Jinrong Hu

**Affiliations:** 1 Key Laboratory of Physical Geography and Environmental Processes, College of Geographical Science, Qinghai Normal University, Xining, China; 2 Key Laboratory of Tibetan Plateau Land Surface Processes and Ecological Conservation (Ministry of Education), Qinghai Normal University, Xining, China; 3 Academy of Plateau Science and Sustainability, Qinghai Provincial People’s Government–Beijing Normal University, Xining, China; 4 College of Life Sciences, Zhejiang Normal University, Jinhua, China; 5 School of Computer Science, Qinghai Normal University, Xining, China; Chinese Academy of Sciences, CHINA

## Abstract

The Sanjiangyuan region, located in the hinterland of the Qinghai-Tibet Plateau(QTP), is highly sensitive to global climate change. Reconstructing its Holocene January and July temperatures is crucial for studying climate change and guiding ecological conservation in alpine regions. Current research on paleoclimate changes in Sanjiangyuan region primarily focuses on small subregions, limiting holistic understanding of regional climate.This study utilizes fossil pollen data, for the first time, integrates the Dynamic Multi-proxy Fusion and Scaling(DMFS) model to reconstruct the Holocene January and July temperature change sequences, thereby exploring temperature variations in the Sanjiangyuan region during 12.5 ka BP. The results indicated:12.5-6.0 ka BP: Both January and July temperatures showed a gradual increase,marking climatic improvement. 6.0-4.0 ka BP, both January and July temperatures remained at high levels, despite their fluctuations. During this period, temperatures reached their peak, reflecting a warm, humid, and most hospitable climate.4.0-2.5 ka BP: Both January and July temperatures showed declining trends to varying degrees, the climate became cold and dry. Post 2.5 ka BP: Both January and July temperatures rebounded. Comparisons with other high-resolution environmental records from the QTP confirmed consistent trends and synchronic dry-wet events. This study contributed essential fossil pollen data and paleotemperature records to the Sanjiangyuan region. This will fill a critical gap in paleoclimate research for the Sanjiangyuan region and provide valuable insights for long-term paleoclimate studies.

## 1. Introduction

Climate change studies on Long-term are a prerequisite for understanding the characteristics and patterns of climate change in a natural context [1]. The Holocene climate more closely resembles modern climatic conditions than other geological periods, which makes it a critical period for studying global climate change [2]. Studying Holocene climate change helps achieve deeper insights into Earth’s climate system, a mechanistic understanding of climate change, improved future climate projections, and the development of ecological conservation strategies [3–5].

Holocene temperature changes changes are characterized by considerable spatial heterogeneity globally. The timing of temperature variations differs across monsoon-dominated regions. Recent research on Holocene temperatures has focused on the seasonal and spatial complexity of the Holocene Thermal Maximum (HTM). Influenced by precession, the HTM was more pronounced during Northern Hemisphere summers, with numerous studies indicating stronger summer solar radiation than present. In contrast, winter temperatures remained lower because of diminished solar radiation. Spatial distribution revealed different models of climate change between high and low latitudes: tropical regions experienced relatively muted temperature changes, whereas the climate in high-latitude areas—affected by ice sheet melting, sea-ice feedback, and other processes—underwent earlier and more significant warming, contributing to a gradual rise in global temperatures. Although this new understanding has gained broad acceptance. However, relevant research still faces challenges due to the uneven global distribution of proxy data and the dependence on the quality and quantity of input data. Therefore, enhancing data accuracy and innovating quantitative reconstruction methods remains a core issue in the temperature reconstruction process.

The Qinghai-Tibet Plateau (from here on, if not specifically stated, Qinghai–Tibet Plateau referring to QTP) possesses a unique Alpine Ecosystem, making it highly sensitive to global climate change [6]. It is a hotspot for researching global climate change.The driving mechanisms of Holocene temperature changes on the QTP unique distinct high-altitude regional characteristics and linkages to the global climate system. Factors such as variations in solar radiation, adjustments in Earth’s orbital parameters [7], ice-albedo feedback [8], interactions between the East Asian monsoon and westerlies [9], and the plateau’s own circulation systems [10] have all exerted varying degrees of influence on Holocene temperature changes across the plateau. The Sanjiangyuan region is situated in the transitional zone between the East Asian and Indian monsoons, where climate is significantly influenced by monsoon dynamics [11]. While numerous recent studies have utilized fossil pollen for paleoclimatic research on the QTP [11–18], most focus on single-sampling site climate records or small-scale paleoclimate reconstructions within the Sanjiangyuan region. Currently, a regional-scale paleoclimate study that covers the entire Three-River Source Region and possesses a long-time series is still very scarce. This study, for the first time, employed the Dynamic Multi-proxy Fusion and Scaling (DMFS) model to reconstruct regional temperatures.The model’s advantage is ability to quantify the differential contributions of individual fossil pollen sites to regional temperature and to establish robust relationships between these fossil pollen sites and the regional climate field. The fundamental principle is that different vegetation types (represented by their pollen) have distinct response thresholds and sensitivities to climatic conditions during the growing season (summer) and non-growing season (winter). By analyzing specific indicator taxa within pollen assemblages (such as cold- or drought-tolerant species), the model establishes a quantitative transfer function with modern observed seasonal temperatures. This process enabled researchers to obtain seasonal temperature signals from the fossil pollen data. Finally, reconstructed the temperature for the Sanjiangyuan region by integrating the temperature estimates from individual sampling sites, and weighted according to their differential contributions to the regional climate.

In view of this, our study aims to enhance the quality and quantity of fossil pollen data in the Sanjiangyuan region and employs a novel temperature reconstruction model to investigate the Holocene of January and July temperature variability in this area. Furthermore, we seek to understand how climate forcing mechanisms differentially affect various regions. The findings are expected to provide valuable references for research on regional climate change and future climate projections in this area.

## 2. Research area overview

**Fig 1:**
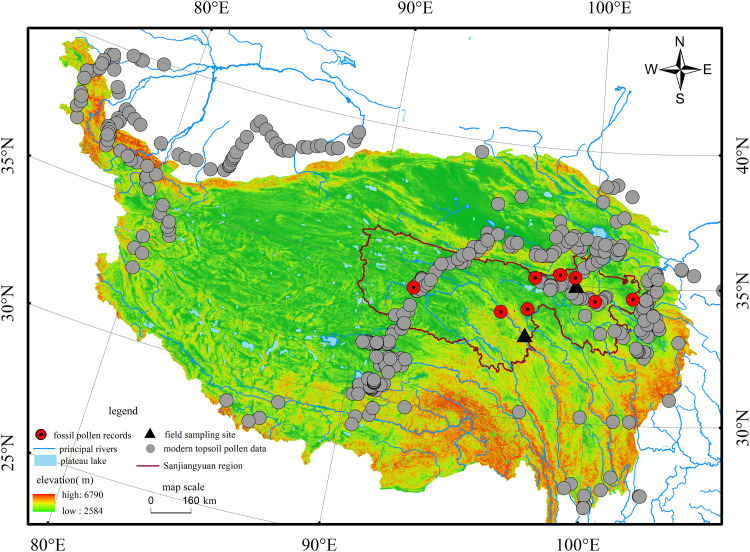
Distribution of Pollen Sites in the Qinghai-Tibetan Plateau and Adjacent Areas, and Location Map of the Study Area. (Zhang, **Y.** (2019). Integration dataset of Tibet Plateau boundary (TPBoundary_HF). National Tibetan Plateau/ Third Pole Environment Data Center. https://doi.org/10.11888/Geogra.tpdc.270099. https://cstr.cn/18406.11.Geogra.tpdc.270099.).

Sanjiangyuan Region located in the core area of the QTP, covers approximately 397,000 km² (accounting for about 10% of the entire plateau), With an average elevation of 4,200 meters [19]. The Yangtze River, Yellow River, and Lancang River(Mekong River) originates from this area (Fig 1). The Sanjiangyuan Region supplies over 60 billion cubic meters of high-quality freshwater resources annually to downstream areas. As China’s and Asia’s the most important water conservation and supply area, it is acclaimed as the “China Water Tower” and “Asia Water Tower”. The region is characterized by a typical plateau continental climate. The mean annual temperature ranges from −5.6°C −3.8°C with significant seasonal variations. Annual precipitation falls between 262.2-772.8 mm, and is primarily concentrated in the summer(data source:MaBingran. et al.2020. https://www.sciencedirect.com/science/article/abs/pii/S0301479720302577) Climatic features include long and severely cold winters, short cool summers, intense solar radiation, and frequent high winds [20]. The region has complex and diverse terrain that is primarily mountainous. Alpine meadow soil is the main soil type, with extensive distributions of permafrost and marshlands. The study area contains nine vegetation types: coniferous forest, broad-leaved forest, mixed coniferous-broadleaved forest, shrubland, meadow, grassland, marsh and aquatic vegetation, cushion vegetation, and sparse vegetation [21]. Among these vegetation types, alpine steppe and alpine meadow serve as the primary vegetation types.

## 3. Data sources and research methods

### 3.1. Profile and sample collection

#### 3.1.1. Xia Dawu profile.

The Xiadawu profile (35.01°N, 99.26°E, elevation 3,988 m) is situated on the terrace of the western bank of the Qingshui River, southwest of Xiadawu Town, Maqên County, Golog Tibetan Autonomous Prefecture, Qinghai Province (Fig 1). The profile has a total depth of 124 cm, stratified as follows:0–22 cm is the topsoil layer, The soil is brownish-gray in color, has a loose texture, exhibits crumb structure, and contains abundant plant roots. 22–104 cm is the loess layer, The soil layer is pale yellow in color, with a denser and more uniform texture, and lacks horizontal stratification. This layer yielded microblades, flakes, scrapers, and fragmented animal bones, all indicative of human activity. At a depth of 103 cm, a distinct black ash layer exists, which exhibits clear signs of combustion and contains incompletely burned carbonized soil and charcoal fragments.104-124 cm is the gravel layer.A total of 62 samples were collected at 2-cm intervals. Additionally, six charcoal fragments for radiocarbon dating were extracted at depths of 30–40 cm, 57 cm, 80–90 cm, 97.5 cm, 105 cm, and 112 cm.

#### 3.1.2. Zhongda profile.

The Zhongda profile (33.24°N, 97.02°E, elevation 3,579 m) is situated on the second terrace of the Tongtian River with in Zhongda Town, Chindu County, Yushu Tibetan Autonomous Prefecture, Qinghai Province (Fig 1). The profile has a total depth of 220 cm, stratified as follows: 0–50 cm is the topsoil layer, The soil contains abundant plant roots and gravel fragments, exhibits a yellowish coloration due to fluvial processes, and contains a small amount of fine sand particles. 50–130 cm is the dark loessial soil layer, It is divided into two sub-layers. At approximately 90 cm depth, there exists 2–3 cm thick interlayer containing sand and gravel fragments. The upper soil layer exhibits a light black color with a uniform texture, indicating relatively poor soil development. In contrast, the lower layer displays a dark black color and shows better soil development.130-210 cm is the loess layer, shows a light yellow color and has a uniform texture. At approximately 200 cm depth, there is a fine sand layer about 5 cm thick. Below 210 cm is a gravel layer, which contains numerous angular gravels. A total of 123 samples were collected at 2-cm intervals. We collected four OSL dating samples at depths of 56, 125, 182, and 210 cm below the surface.

### 3.2. Data sources

This study utilizes fossil pollen data, modern topsoil pollen data, and instrumental climate records (Table 1).

**Table 1:** Data types and sources.

Data types	Data sources
Modern topsoil pollen data	Modern topsoil pollen data from 499 sites across the QTP and adjacent regions, National Standardized Modern Pollen Database (1980–2020) Pollen Dataset Climate Research Dataset (https://www.geodata.cn/data/datadetails.html?), Topsoil pollen data from the Northeastern Qinghai-Tibet Plateau [22]
Fossil pollen data	Kuhai drilling core [23], Koucha Lake drilling core [24], Maqin profile [25], Donggi Cona profile [26], Canxiong Gasu profile [27], Gaqing profile [28], Ngoring Lake Profile [29], and Bande Lake sediment core BDH19A [30]
Modern instrumental climate data	1km monthly mean temperature dataset for China (1901–2023), National Qinghai-Tibet Plateau Data Center [31] (https://doi.org/10.11888/Meteoro.tpdc.270961)

### 3.3. Research methods

#### 3.3.1. Pollen analysis.

**Fig 2:**
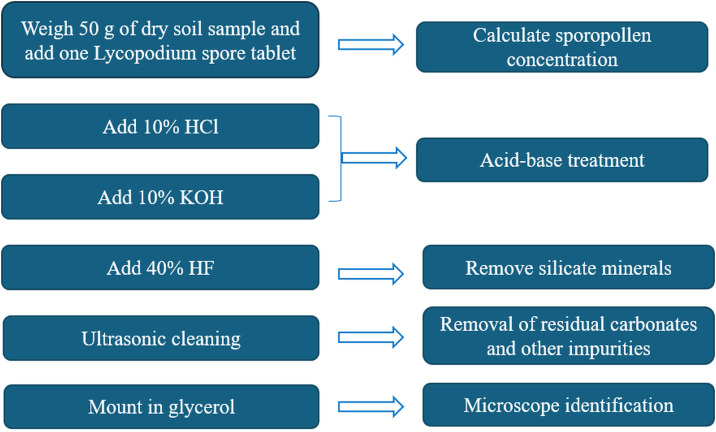
Pollen experimental pretreatment process.

We processed the pollen samples from the Xiadawu and Zhongda profile (Fig 2). After that, we identified pollen grains with an optical biological microscope at magnifications of ×400 and ×1000 magnification, and referring to published palynological atlases [32]. The pollen sample pre-treatment and identification were conducted at the Key Laboratory of Qinghai Normal University and the Qinghai Institute of Salt Lakes, Chinese Academy of Sciences. Six charcoal fragments samples from the Xia Dawu profile were sent to the Accelerator Mass Spectrometry (AMS) Laboratory of the Quaternary Dating Laboratory at Peking University for AMS ^14^C dating. The obtained ^14^C ages were calibrated to calendar years using the IntCal20 tree-ring calibration curve in the OxCal -University of Oxford software [33]. Four OSL dating samples from the Zhongda profile were processed at the OSL Laboratory of the Key Laboratory of Qinghai Normal University.

#### 3.3.2. Transfer function method.

The reconstruction principle of the transfer function method [34]. In actual operation, the weighted averaging partial least squares (WA-PLS) method implemented in the Untitled-C2 software was employed to reduce the marginal effects of the transfer function. Paleotemperature records for January and July were reconstructed from ten pollen sites after achieving a confidence level (R²) exceeding 0.85, including: Xia Dawu profile,Zhongda profile, Kuhai drilling core [23], Koucha Lake drilling core [24], Maqin profile [25], Donggi Cona profile [26], Canxiong Gasu profile [27], Gaqing profile [28], Ngoring Lake Profile [29], and Bande Lake sediment core BDH19 [30].

#### 3.3.3. Establishment of the Dynamic Multi-proxy Fusion and Scaling model (DMFS).

The study extracted January and July mean temperature data for the period 1901–1943 (32 years) from 10 pollen sampling sites and the Sanjiangyuan region respectively using ArcGIS software. The data obtained from ArcGIS were organized and imported into SPSS version 26. The study established a linear regression model between 10 fossil pollen sampling sites and the regional January mean temperature by iteratively integrating their relationships, ultimately consolidating it into the DMFS-1 (January) temperature model.The DMFS-7 (July) temperature model was constructed following the same methodology.

The DMFS method for the first time quantifies the differential contributions of various fossil pollen sampling sites, establishing a robust relationship between them.

This method is particularly well-suited for analyzing the highly nonlinear and complex fuzzy relationships between climatic variables and fossil pollen sampling sites in the Sanjiangyuan region. The DMFS approach provides a more advanced and appropriate framework for quantifying the relationship between fossil pollen sites and Sanjiangyuan region of temperature.

## 4. Results analysis

### 4.1. Reliability Analysis of the DMFS

**Fig 3:**
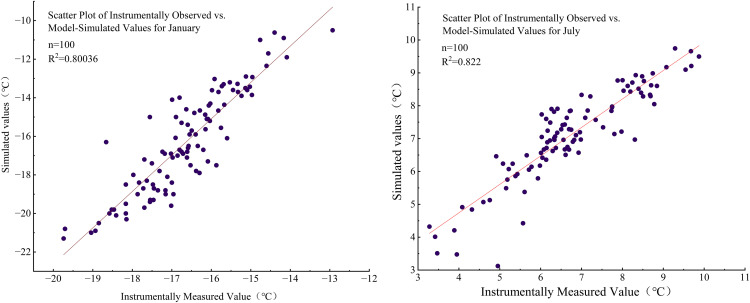
Relationship between instrumental and simulated values of January and July temperatures in the Sanjiangyuan region (1923–2023).

The DMFS model reconstructed the monthly mean temperatures for January and July in the Sanjiangyuan region from 1923 to 2023. We performed a Pearson correlation analysis between the simulated and modern instrumental values for January and July mean temperatures. The results showed that the coefficients of determination (R²) were 0.80 and 0.82, respectively, and both were statistically significant at the 0.01 level (two-tailed test) (Fig 3). This demonstrates high credibility of the DMFS-1 and DMFS-7 models in reconstructing January and July mean temperatures for the Sanjiangyuan region. However, due to incomplete chronological coverage of some fossil pollen records. For example: Xia Dawu profile, Donggi Cona profile [26], Gaqing profile [28], Therefore, the Long Short-Term Memory (LSTM) method was employed to perform long-term temporal interpolation of the reconstructed temperature data, thereby completing the January and July mean temperature reconstructions. The adopted China 1-km resolution monthly mean temperature dataset (1901–2023), despite its comprehensiveness and detail, still proves insufficient in spatial resolution for regional-scale microclimate analysis. If longer time series of fossil pollen records and higher-resolution modern instrumental climate data could be acquired in the future, this would significantly enhance the reliability of the reconstruction.

### 4.2. AMS ¹⁴C and OSL dating results

**Fig 4:**
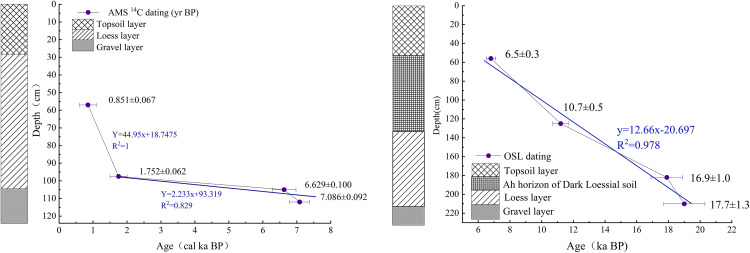
Age-Depth Relationship of the Xia Dawu Profile (left) and Zhongda Profile (right). (In the formula, X denotes Age; Y denotes Depth; the columnar diagram represents stratigraphic characteristics of the profiles).

Calibrated ages for the AMS ^14^C samples from the Xia Dawu profile are presented in Table 2. Sample XDW2–6 (80–90 cm depth) exhibited an age inversion and was therefore excluded from the dataset. We discovered microblades, flakes, scrapers, and fragmented animal bones within the loess layer at 23–104 cm depth in the Xia Dawu profile, all indicating past human activity. At a depth of 103 cm, a black ash layer was present. This layer exhibited clear signs of burning and contained well-preserved burnt soil and charcoal fragments.Therefore,We preliminarily attribute this stratigraphic age reversal to anthropogenic disturbances. Chronological information of OSL Samples from the Zhongda Profile (Table 3). The dating results indicate that ages increase with sample depth, consistent with stratigraphic deposition principles. A linear relationship was applied to fit the depth-age correlation for both the Xia Dawu and Zhongda profiles, and depths were converted to ages using interpolation and extrapolation methods (Fig 4).

**Table 2:** Chronostratigraphic ages for the XDW2 profile.

Sample ID	Dating lab number	Depth (cm)	Material	AMS ^14^C dating (yr BP)	Calibrated age (cal yr BP)
XDW1−1	BA131374	30-40	Charcoal	Modern carbon	0
XDW1–2	BA131375	57	Charcoal	0.92 ± 0.025	0.846 + 0.047
XDW1–3	BA131376	97.5	Charcoal	1.79 ± 0.025	1.659 + 0.037
XDW1–4	BA131377	105	Charcoal	5.82 ± 0.035	6.629 + 0.055
XDW1–5	BA131378	112	Charcoal	6.195 ± 0.03	7.679 + 0.56
XDW1–6	BA131379	80-90	Charcoal	5.375 ± 0.03	6.193 + 0.078

**Table 3:** Chronostratigraphic ages for the Zhongda profile.

Sample ID	Depth (cm)	U(ppm)	Th(ppm)	K(%)	Dose rate (Gy·ka ⁻ ^1^)	Equivalent dose (Gy)	Age (ka BP)
ZD-OSL-1	56	2.49 ± 0.4	12.25 ± 0.7	1.76 ± 0.04	3.39 ± 0.08	21.94 ± 0.93	6.5 ± 0.3
ZD-OSL-2	125	2.56 ± 0.4	13.30 ± 0.7	1.96 ± 0.04	3.66 ± 0.08	39.28 ± 1.29	10.7 ± 0.5
ZD-OSL-3	182	2.70 ± 0.4	11.82 ± 0.7	1.84 ± 0.04	3.44 ± 0.08	58.05 ± 3.00	16.9 ± 1.0
ZD-OSL-4	210	1.79 ± 0.3	8.58 ± 0.6	1.39 ± 0.03	2.92 ± 0.07	51.57 ± 3.60	17.7 ± 1.3

### 4.3. Characteristics of the fossil Pollen Spectrum in the Xia Dawu Profile

**Fig 5:**
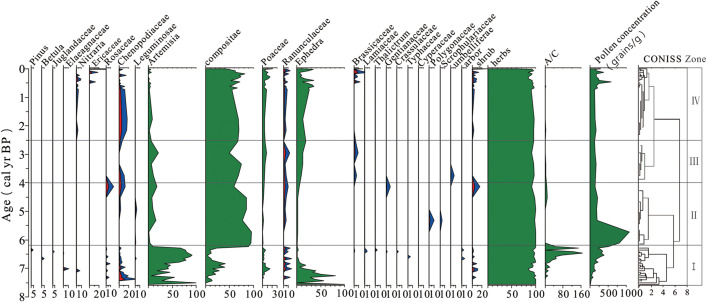
Characteristics of the sporo-pollen assemblage in the Xia Dawu Profile (The blue section is magnified 3×).

Electron microscopic examination identified 23 distinct pollen taxa from 51 fossil pollen samples in the Xia Dawu profile. Arboreal pollen three types: *Pinus*, *Betula*, Juglandaceae. Shrub pollen Seven types: Elaeagnaceae, *Nitraria*, Ericaceae, Rosaceae, Chenopodiaceae, *Ephedra*, Fabaceae. Herbaceous pollen thirteen types: *Artemisia*, Compositae, Poaceae, Ranunculaceae, Brassicaceae, Lamiaceae, *Thalictrum*, Gentianaceae, Crassulaceae, Typhaceae, Polygonaceae, Scrophulariaceae, Apiaceae. The pollen diagrams were plotted using Tilia and Tilia-Graph software [35] (Fig 5).

Stage I (7.5-6.2 cal yr BP) *Artemisia*, Compositae, and *Ephedra* showed high content, with ranges of (0–91.5%, mean 53.5%), (0–41.5%, mean 15.6%), and (0–55%, mean 22.7%). These were followed by Raunculaceae, Chenopodiaceae, Poaceae. *Pinus*, *Betula*, and Juglandaceae was occasionally encountered.Terrestrial pollen concentration (3.5–385.5 grains/g mean: 136.6 grains/g).

Stage Ⅱ (6.2-4.0 cal yr BP) Compositae exhibited the highest content, ranging (86.0–96.5%, mean 88.1%). It was followed by *Artemisia*, *Ephedra*. Ranunculaceae, Poaceae Content below 1%. Polygonaceae, Scrophulariaceae, and Fabaceae was occasionally encountered.Terrestrial pollen concentration (170.8–1090.6 grains/g mean: 495.6 grains/g).

Stage Ⅲ (4.0-2.5 cal yr BP) was dominated by Compositae (50.0-75.5%, mean 64.2%), *Artemisia* (5.8-21.5%, mean 12.4%). *Ephedra* (5.32-18.60%, mean 11.66%). In the next place:In the next place were Poaceae, Chenopodiaceae, Ranunculaceae. Gentianaceae, Apiaceae Content were below 1%. Terrestrial pollen concentration (125.03-168.27 grains/g mean: 143.37 grains/g).

Stage Ⅳ (2.5-0 cal yr BP) was dominated by Compositae (41.24-82.76%, mean 62.59%), *Artemisia* (0-8.14%, mean 2.54%), Poaceae (2.91-20.62%, mean 7.50%). Accompanied by minor amounts of Ranunculaceae, Chenopodiaceae, Cruciferae, *Nitraria*, and Ericaceae. Terrestrial pollen concentration (124.36–578.25% mean: 223.56% grains/g).

### 4.4. Characteristics of the fossil sporo-pollen assemblage in the Zhongda Profile

**Fig 6:**
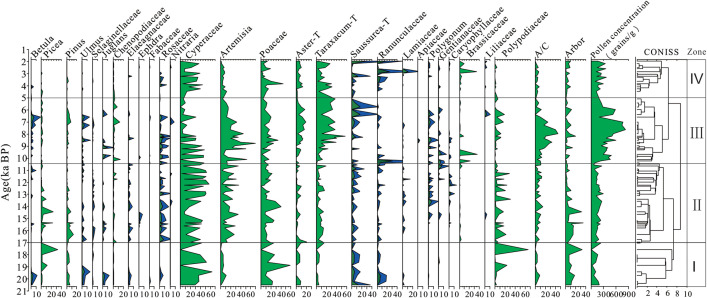
Characteristics of the sporopollen assemblage in the Zhongda Profile (Blue section magnified 3×).

The microscopic analysis of 81 samples from the Zhongda Profile identified a total of 25 pollen taxa. Arboreal pollen six types: *Betula*, *Pinus*, Juglandaceae, Ulmus, *Picea*, Selaginellaceae. Shrub pollen six types: Elaeagnaceae, *Ephedra*, Chenopodiaceae, Rosaceae, Fabaceae and *Nitraria*. Herbaceous thirteen types: *Artemisia*, Compositae, Poaceae, Ranunculaceae, Brassicaceae, Gentianaceae, Polygonaceae, Lamiaceae, Cyperaceae, Apiaceae, Liliaceae, Caryophyllaceae, and Polypodiaceae. The pollen diagrams were plotted using Tilia and Tilia-Graph software [35] (Fig 6).

Stage I (20.6–17.0 ka BP) was dominated by Cyperaceae (0–63.94%, mean: 33.41%) and Poaceae (0–58.82%, mean: 21.12%). followed by Polypodiaceae, *Picea*, *Artemisia*, *Asteraceae*, *Taraxacum* and *Pinus*.The pollen assemblage contained trace amounts of Betula, Ranunculaceae, Juglandaceae, Rosaceae, Selaginellaceae, and Elaeagnus. Terrestrial pollen concentration (27.40-230.06 grains/g mean:139.14 grains/g).

Stage Ⅱ (17.0–10.4 ka BP) was dominated by Cyperaceae (2.75–57.49%, mean: 31.45%), Poaceae (0.47–40.95%, mean: 21.12%), *Artemisia* (2.93–36.70%, mean: 15.65%), and Polypodiaceae (0–31.07%, mean: 7.81%). Followed by *Pinus*, *Picea* and *Taraxacum*. Minor taxa include *Asteraceae*, Rosaceae, Polygonaceae, Chenopodiaceae, and Caryophyllaceae were also observed. Terrestrial pollen concentration(101.45–296.91 grains/g mean: 177.49 grains/g).

Stage Ⅲ(10.4-5.0 ka BP) Cyperaceae (0–57.85%, mean: 16.28%), Poaceae (1.89–34.09%, mean: 47.85%), *Artemisia* (3.64–69.5%, mean: 24.58%), *Taraxacum* (1.40–57.75%, mean: 21.63%) constituted the dominant components. Followed by *Asteraceae*, Chenopodiaceae, Polypodium, Brassicaceae, Rosaceae, *Pinus*, Ranunculaceae, Polygonaceae. Terrestrial pollen concentration (101.45–296.91 grains/g mean: 177.49 grains/g), reaching the maximum level recorded for the entire period.

Stage Ⅳ(5.0-1.95 ka BP) was dominated by Cyperaceae (0–44.44%, mean: 21.61%) and Poaceae (1.11–45.02%, mean: 11.92%). *Artemisia*, *Taraxacum*, *Asteraceae* and Ranunculaceae occurred at relatively low percentages. The average percentage content of *Betula*, *Pinus*, *Picea*, Chenopodiaceae, Fabaceae, Elaeagnaceae, Rosaceae, Lamiaceae, Polygonaceae, Gentianaceae, and Polypodiaceae were all below 1%. Terrestrial pollen concentration (100.65–373.33 grains/g mean: 213.99 grains/g).

### 4.5. Multicollinearity test for January and July temperatures at 10 fossil pollen sampling sites in the Sanjiangyuan region

Multicollinearity tests for January and July temperatures at 10 fossil pollen sampling sites revealed that all Tolerance (TOL) values exceeded 0.1. By applying the Variance Inflation Factor (VIF), variables containing redundant information were eliminated.The process continued iteratively until all Variance Inflation Factor (VIF) values fell below the threshold of 5 [36], indicating that the information overlap between variables had been minimized. Consequently, as shown in (Tables 4 and 5), the entirety has met the inspection standards for Holocene paleotemperature reconstruction in the Sanjiangyuan region.

**Table 4:** (TOL) and (VIF) diagnostic metrics for January temperatures at 10 fossil pollen sampling sites.

	Xia Dawu Profile	Canxiong Gasu profile	Gaqing profile	Zhongda Profile	Donggi Cona profile	Koucha Lake drilling core	Kuhai drilling core	Maqin profile	Ngoring Lake Profile	Bande Lake sediment core BDH19A
TOL	0.411	0.542	0.731	0.647	0.579	0.508	0.425	0.297	0.830	0.811
VIF	2.432	1.843	1.368	1.545	1.726	1.969	2.354	3.369	1.183	1.233

**Table 5:** (TOL) and (VIF) diagnostic metrics for July temperatures at 10 fossil pollen sampling sites.

	Xia Dawu Profile	Canxiong Gasu profile	Gaqing profile	Zhongda Profile	Donggi Cona profile	Koucha Lake drilling core	Kuhai drilling core	Maqin profile	Ngoring Lake Profile	Bande Lake sediment core BDH19A
TOL	0.398	0.605	0.450	0.464	0.401	0.523	0.638	0.592	0.744	0.850
VIF	2.514	1.653	2.224	2.165	2.496	1.911	1.568	1.690	1.344	1.117

**Fig 7:**
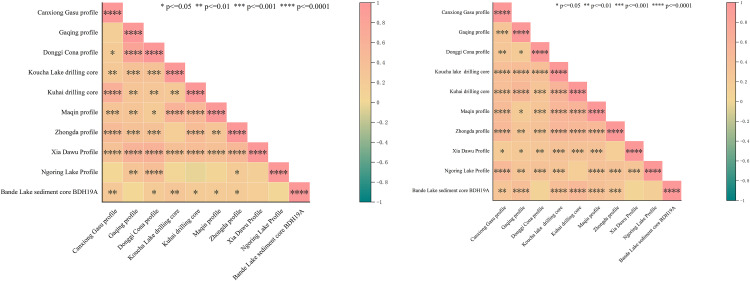
Correlation test of January (left) and July (right) temperatures across 10 fossil sporopollen sampling sites.

The collinearity diagnostics analysis of 10 fossil pollen sampling sites in the Sanjiangyuan region indicated that the pollen assemblage was positively correlated with both January and July mean temperatures (Fig 7). The goodness-of-fit (R²) exhibited progressive enhancement with the increase in fossil pollen sampling sites, indicating a continuous strengthening of the regression equation’s robustness.

Consequently, the models achieved higher accuracy in simulating January and July mean temperatures, ultimately yielding the definitive model (DMFS-1) and (DMFS-7) in (Tables 6 and 7).

**Table 6:** Construction of the DMFS-_1_ regression model.

Accumulated fossil pollen sampling sites	January mean temperature model	R	R^2^
C	T_1_ = 0.789C-1.515	0.811	0.657721
C, G	T_1_ = 2.855−0.919*C* + 1.931*G*	0.815	0.664225
C, G, D	T_1_ = 2.297−0.021*C* + 0.503*G* + 0.949*D*	0.829	0.687241
C, G, D, Q	T_1_ = 0.386 + 3.39*C* + 0.615*G* + 1.517*D*-2.472*Q*	0.842	0.708964
C, G, D, Q, K	T_1_ = −0.358−4.117*C* + 0.633*G* + 1.558*D*-2.417*Q*-0.209*K*	0.851	0.724201
C, G, D, Q, K, M	T_1_ = 2.199−0.336*C* + 2.029*G* + 1.464*D*-2.665*Q*-0.796*K* + 1.329*M*	0.884	0.781456
C, G, D, Q, K, M, Z	T_1_ = −4.683−0.332*C* + 0.537*G* + 2.463*D*-2.688*Q*-0.808*K* + 1.382*M*-0.085*Z*	0.897	0.804609
C, G, D, Q, K, M, Z, X	T_1_ = −7.662−0.283*C* + 1.840*G* + 1.131*D*-2.67*Q*-1.201*K* + 0.731*M*-0.216*Z* + 1.103*X*	0.903	0.815409
C, G, D, Q, K, M, Z, X, E	T_1_ = −7.711−0.285*C* + 2.339*G* + 1.136*D*-2.67*Q*- 1.201*K* + 0.733*M*-0.217*Z* + 1.102*X*-0.737*E*	0.928	0.861184
C, G, D, Q, K, M, Z, X, E, R	DMFS-_1_ = −7.516−0.289*C* + 0.295*G* + 1.179*D*-2.602*Q*-1.086*K* + 0.765*M*-0.121*Z* + 1.109*X* + 0.79*E*-0.313*R*	0.949	0.900601

C: Canxiong Gasu profile [27], G: Gaqing profile [28], D: Donggi Cona profile [26], Q: Koucha Lake drilling core [24], K: Kuhai drilling core [23], M: Maqin profile [25], Z: Zhongda Profile, X: Xia Dawu Profile, E: Ngoring Lake Profile [29] R: Bande Lake sediment core BDH19A [30].

**Table 7:** Establishment of the DMFS-7 regression model.

Accumulated fossil pollen sampling sites	July mean temperature model	R	R^2^
C	T_7_ = 4.580 + 0.201*C*	0.785	0.616225
C, G	T_7_ = 3.751−0.16*C* + 0.956*G*	0.793	0.628849
C, G, D	T_7_ = 5.131 + 0.259*C* + 0.444*G*-0.438*D*	0.819	0.670761
C, G, D, Q	T_7_ = 2.512 + 0.383*C*-0.198*G*-0.818*D* + 1.671*Q*	0.823	0.677329
C, G, D, Q, K	T_7_ = 4.824 + 0.477*C*-0.029*G*-0.864*D* + 1.894*Q*-0.820*K*	0.827	0.683929
C, G, D, Q, K, M	T_7_ = 5.301 + 0.464*C* + 0.013*G*-0.796*D* + 1.944*Q*-0.725*K*-0.422*M*	0.841	0.707281
C, G, D, Q, K, M, Z	T_7_ = 3.138 + 0.364*C*-0.232*G*-1.178*D* + 1.865*Q* + 0.155*K*-0.264*M* + 0.831*Z*	0.856	0.732736
C, G, D, Q, K, M, Z, X	T_7_ = 4.738 + 0.435*C*-0.031*G*-0.969*D* + 1.885*Q*-0.792*K*-0.4.2*M* + 0.896*Z* + 0.206*X*	0.884	0.781456
C, G, D, Q, K, M, Z, X, E	T_7_ = 15.820 + 0.22*C* + 0.207*G* −0.968*D* + 1.37*Q*-0.923*K* + 0.184*M* + 1.075*Z*-0.05*X*-1.316*E*	0.897	0.804609
C, G, D, Q, K, M, Z, X, E, R	DMFS-_7_ = 15.283 + 0.187*C* + 0.204*G*-0.95*D* + 1.348*Q*-0.918*K* + 0.158*M* + 1.087*Z* + 0.048*X*-1.312*E* + 0.105*R*	0.919	0.844561

C: Canxiong Gasu profile [27], G: Gaqing profile [28], D: Donggi Cona profile [26], Q: Koucha Lake drilling core [24], K: Kuhai drilling core [23], M: Maqin profile [25], Z: Zhongda Profile, X: Xia Dawu Profile, E: Ngoring Lake Profile [29], R: Bande Lake sediment core BDH19A [30].

### 4.6. Integrated Holocene January and July temperature reconstructions from Fossil pollen sites in the Sanjiangyuan region

**Fig 8:**
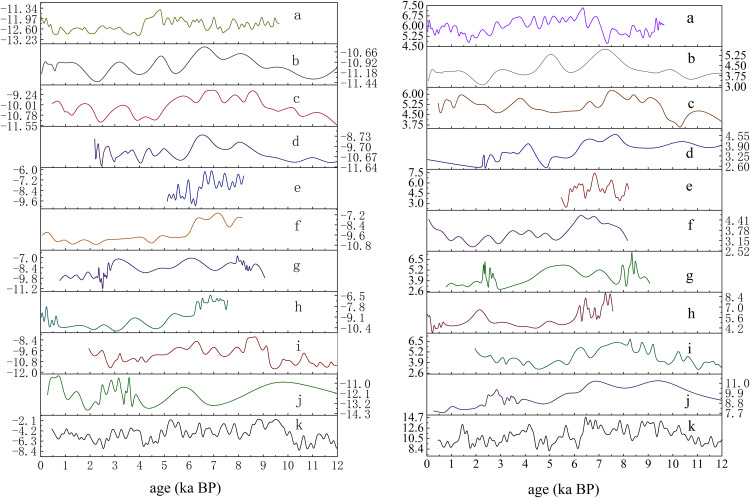
Reconstructed January (left) and July (right) Mean Temperatures from 10 fossil Pollen Sites (Unit:°C). **(a)** Bande Lake sediment core BDH19A [30]; **(b)** Maqin profile [25]; **(c)** Kuhai drilling core [23]; **(d)** Koucha Lake drilling core [24]; **(e)** Donggi Cona profile [26]; **(f)** Gaqing profile [28]; **(g)** Canxiong Gasu profile [27]; **(h)** Xia Dawu Profile; **(i)** Zhongda Profile; **(j)** Ngoring Lake Profile [29]; **(k)** Sanjiangyuan region.

Based on sedimentary records from the Bande Lake BDH19A core, Maqin section, Kuhai drill core, Cocha Lake drill core, Donggi Cona section, Gaqing section, Canxionggasu section, Xiada Wu section, Zhongda section, and Ngoring Lake section in the Sanjiangyuan region, The overall trend showed that January and July mean temperatures fluctuated and rose during the early Holocene, peaked in the mid-Holocene, and exhibited a decline-recovery pattern in the late Holocene (Fig 8).

## 5. Discussion

The pollen assemblages in the sediments and temperature of the QTP exhibit significant correlations: the content of arboreal pollen (e.g., *Picea*/*Abies*) show a negative correlation with mean annual temperature, while herbaceous indicators such as the *Artemisia*/Chenopodiaceae ratio (A/C) exhibit a positive correlation with summer temperature. In the monsoon region, fern spores are closely related to accumulated temperature, whereas in the westerly region, *Ephedra* pollen shows an exponential relationship with annual temperature range. High abundances of *Ephedra*, *Nitraria*, and Chenopodiaceae may primarily indicate arid conditions, while high abundances of Ericaceae and Gentianaceae are more indicative of cold and humid environments. Cyperaceae, as the dominant pollen of alpine meadows, typically show cold and wet conditions when abundant. *Artemisia* is often associated with relatively warm and dry conditions. The Chenopodiaceae family, characterized by its tolerance to drought and salinity, usually indicates warm and arid environments when highly abundant.

### 5.1. Analysis of fossil pollen assemblage characteristics of the Xiada Wu profile

Stage I (7.5–6.2 cal yr BP) was dominated by herbaceous vegetation, with terrestrial fossil pollen concentration (average 136.6 grains/g). The percentage content of arboreal and shrub pollen was higher during this period than in other stages, indicated an improvement in the climate environment. Pollen records from the Ye Zhi Ze SKJ and JTL profile [37] (11.0–5.0 ka BP) and the Da Lian Hai site(9.4–3.9 ka BP) [38] both indicated a climatic improvement. Sediments from the Qilian profile and Huang Yang He profile showed a warm and humid climate from 8.0–6.0 ka BP [39]. The pollen records from the Xiada Wu profile corresponded well with other high-resolution environmental records during this stage.

Stage II (6.2–4.0 cal yr BP): Terrestrial fossil pollen concentration (average 495.6 grains/g) reached the highest value among the four stages, indicating a warm and humid climate environment. 7.0–4.0 ka BP, lake productivity in Ximen Co significantly increased [40], The Holocene temperature reconstructions from the Yaoxian and Jingchuan loess profiles on the Loess Plateau showed the highest temperatures during [41]. These high-resolution environmental records were consistent with the pollen assemblage characteristics of the Xiada Wu profile during this period.

Stage III (4.0–0.5 cal yr BP): The terrestrial fossil pollen concentration and the content of Compositae and *Artemisia* decreased significantly, while the content of *Ephedra* increased, indicating a deterioration of the climate. 4.5–1.0 ka BP, the degree of peat humification in Hongyuan decreased [42] 4.5–0.5 ka BP, ACL in the Zhangwuzhai profile was high and even reached their maximum values, indicating poor climate conditions [43]. The environmental changes indicated by the pollen records from the Xiada Wu profile were consistent with those indicated by other environmental proxy indicators.

### 5.2. Characteristic analysis of fossil pollen assemblages from the Zhongda profile

Stage I (20.6-17.0 ka BP),terrestrial fossil pollenn concentration (average 139.4 grains/g), with arboreal pollen averaging 12.13%. This assemblage was dominated by *Artemisia* and Poaceae, with a minor presence of conifers *(Picea* and *Pinus*), showed a high degree of consistency with the pollen assemblage from the RM core in the Zoige Basin (20-17.0 ka BP) [44].

Stage II (17.0–10.4 ka BP), terrestrial fossil pollenn concentration (average 177.49 grains/g), with the emergence of *Ephedra* indicating aridity. The high-resolution record from the Qinghai Lake QH-2000 core [45] indicated that a cold-dry phase(16.0–15.2 ka BP), characterized by minimal pollen content. Subsequently, the climate improved during the period of 15.2–10.4 ka BP, which was marked by a continuous increase in arboreal and herbaceous pollen concentrations, exceeding modern levels.

Stage III (10.4–5.0 ka BP), terrestrial fossil pollenn concentration (average 432.24 grains/g), which reached the highest value in the Holocene, indicating that this period was the peak of warm and humid.The with Chen Co and Zhabuye Salt Lake which showed enhanced early Holocene monsoonal intensity [46,47]; Vegetation succession from alpine steppe to meadow in Luanhaizi Basin (10.0–4.5 ka BP), signaled progressive climatic amelioration [12]. Collectively, this evidence validates the existence of a coherent Holocene climatic optimum in the northeastern of QTP.

Stage IV (5.0-1.95 ka BP): terrestrial fossil pollenn concentration (average 218.25 grains/g) decreased compared to the previous stage, indicating deteriorated climate conditions. The DG03 core from Ga Hai Lake showed that the TOC content in lake sediments was less than 0.7%(4.7 to 0 ka BP). The combination of Si/Al-Fe ratio, CIA and ICV indicated a reduction in surface differentiation intensity and deteriorated climate [48,49]. These high-resolution environmental records were consistent with the fossil pollenn assemblage characteristics of the Zhongda profile.

### 5.3. Integrated reconstruction of January and July mean temperatures during the Holocene in the Sanjiangyuan region

The integrated reconstruction of January and July mean temperatures in the Sanjiangyuan region during the Holocene could be roughly divided into four stages: an upward trend from 12.5 to 6.0 ka BP, relatively high temperatures from 6.0 to 4.0 ka BP, a downward trend from 4.0 to 2.5 ka BP, and a rebound from 2.5 to 0.5 ka BP.

12.5–6.0 ka BP, The reconstruction results and other environmental records together indicated a trend toward a warmer and humid climate.The Zangser Kangri ice core δ¹⁸O [50], summer solar radiation at 30°N [51], Northern Hemisphere temperature reconstruction [1], the warmest month temperature reconstruction of Tiancai Lake Chironomid-inferred [52], Holocene temperature reconstruction in China [53], The Greenland ice core [54] records (such as NGRIP). Existing studies indicated that the upward temperature trend in the early Holocene was attributed to increased solar radiation and changes in the Milankovitch cycles(11.7–6.0 ka BP). The maximum value of summer solar radiation at 65°N occurred between 11.0 and 10.0 ka BP [55]. These indicated that the increase in solar radiation directly drove the melting of ice sheets and global warming. The cold events occurring around 11.0 ka BP and 8.2 ka BP were also very close in their timing.

6.0–4.0 ka BP, the climate was generally warm and the environment was most favorable.The Qinghai Lake QH-2005 sediment core [56] recorded a peak in redness index(10.0–5.3 ka BP). the Greenland ice core [54] (10.0 to 6.0 ka BP), the Northern Hemisphere temperature reconstruction [1] (0.0–5.0 ka BP) and Holocene temperature reconstruction in China [53] (9.5–5.3 ka BP) all reached maximum temperature. These environmental records and reconstruction results collectively indicated temporal disparities in the occurrence of the Holocene Thermal Maximum (HTM).

4.0–2.5 ka BP,The integrated reconstruction of January and July mean temperatures in the Sanjiangyuan region,along with other environmental records, all showed that the climate began to deteriorate. The warmest month temperature reconstruction of Tiancai Lake Chironomid-inferred [52] (6.5–1.5 ka BP), The Asian summer monsoon index from Qinghai Lake weakened(6.5–2.7 ka BP) [57]. The Zangser Kangri ice core δ¹⁸O [50], Northern Hemisphere temperature reconstruction [1], Holocene temperature reconstruction in China [53] and Greenland ice core temperature reconstruction [54] all showed a gradual decline to varying degrees(5.5–2.5 ka BP). The timing of the 4.2 ka BP cold event was also well aligned.

**Fig 9:**
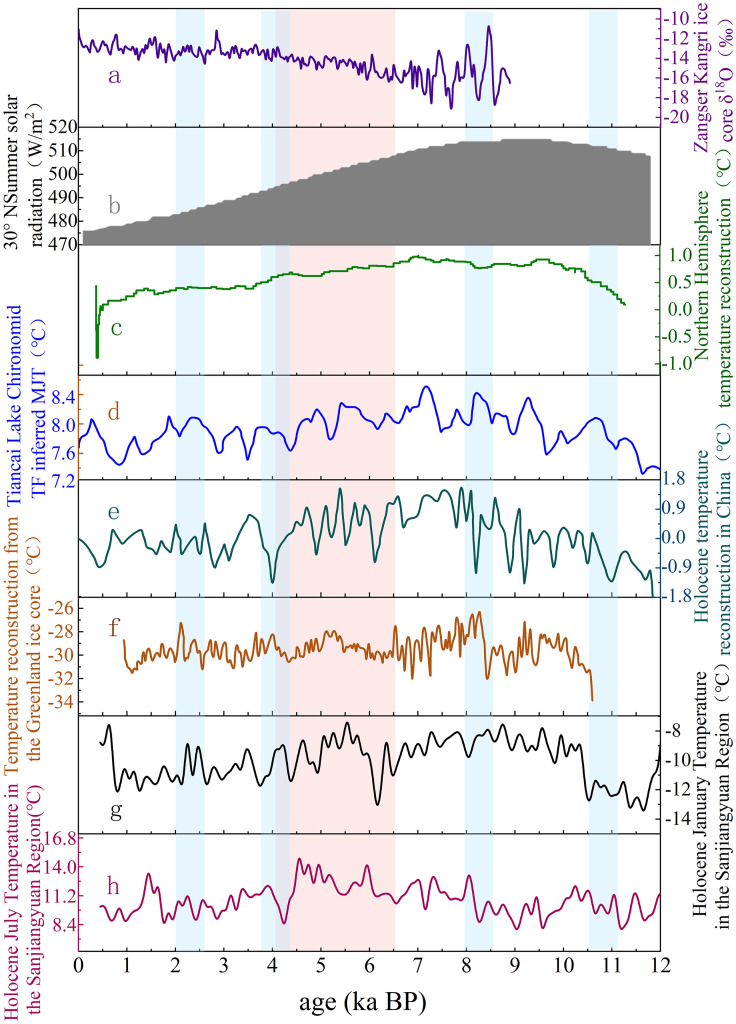
Comparison of integrated Holocene January and July temperature reconstructions in the Sanjiangyuan region with other environmental records. **(a)** The Zangser Kangri δ^18^O record indicates 30-year means [50]; **(b)** Summer solar radiation at 30°N [51]; **(c)** Northern Hemisphere (30°–90°N) temperature reconstruction [1]; **(d)** Tiancai Lake Chironomid-inferred mean July temperatures using a 5 sample running average (~250 year mean) [52]; **(e)** Chinese Holocene temperature reconstruction [53]; **(f)** Greenland ice core temperature reconstruction [54]; **(g)** Sanjiangyuan region Holocene January temperature; **(h)** Sanjiangyuan region Holocene July temperature; Color annotations: Blue shading: Markscold events (e.g., 8.2 ka BP and 4.2 ka BP anomalies); Orange shading: Indicates the Holocene Thermal Maximum (HTM) period (~6.0–4.0 ka BP in Sanjiangyuan). Environment Data Center. https://doi.org/10.11888/Geogra.tpdc.270099. https://cstr.cn/18406.11.Geogra.tpdc.270099).

The reconstructed January and July mean temperature series in the Sanjiangyuan region since the Holocene demonstrated high consistency with other high-resolution environmental records from the QTP and several global high-resolution environmental records (Fig 9). Therefore, the research results have a certain degree of reliability and accuracy. Under the backdrop of global climate change, the climate change across the entire QTP exhibits overall consistency.

The timing of the Holocene Thermal Maximum (HTM) varied significantly across the globe. Generally. In Northern Europe and North America, the HTM occurred between 9.0-6.0 ka BP [58], whereas in Western Europe it spanned from 8.0-5.0 ka BP [59]. In parts of South America, the HTM lasted from 8.0-4.0 ka BP, indicating an extended duration [60]. In the monsoon-influenced regions of China, the HTM occurred between 7.5-5.0 ka BP, about 1−2 millennia later than in Northern Europe, aligning with the period of intensified summer monsoon. On the QTP, the HTM was even more delayed, occurring from 6.0-4.0 ka BP compared to the eastern monsoon regions of China [61]. Multiple proxy records, including pollen assemblages [62],consistently indicated that the HTM on the QTP occurred later than in most other parts of the world.

6.0-3.0 ka BP on the QTP, the enhanced East Asian monsoon [63] and intensified Indian monsoon increased atmospheric moisture transport, leading to thicker cloud cover that amplified downward longwave radiation. This mechanism contributed to 30–40% of the warming magnitude during the mid-Holocene warm period [64]. Additionally, ice-snow melting and vegetation expansion altered surface albedo by reducing reflectivity of solar radiation [65], which also served as a key factor for the climatic warming during this interval.The extensive snow cover over the QTP acts as a critical factor within its climate system. The thick snow accumulated during winter and spring has a high albedo, reflecting a amount of solar radiation back to space. This greatly reduces the amount of heat absorbed by the surface, leading to strong cooling of both the land surface and the lower atmosphere. Furthermore, spring snowmelt consumes considerable energy, which further delays the warming of the surface and the atmosphere.Therefore, even though global environment and solar insolation had already shifted toward warmer climates during the Early-Middle Holocene, the persistent snow-albedo positive feedback mechanism effectively suppressed temperature rise on the QTP, resulting in a lagged thermal maximum. The deep snow cover establishes the QTP as a significant “cold source” in spring,which stands in sharp contrast to its role as a “heat source” in summer, thereby further delaying the overall transition of the regional climate system into a warm phase. Due to its high altitude, the QTP exhibits inherent thermal inertia, requiring a longer time to absorb heat. The slower rate of ice and snow melt, along with the prolonged maintenance of high surface albedo, extended the duration of cooling effects [66]. As a result, the HTM on the QTP occurred later than in the monsoonal regions of China and most other areas worldwide.

## 6. Conclusion

This study integrated fossil pollen data from the Xiadawu and Zhongda profiles with eight additional fossil pollen records from the Sanjiangyuan region, applied the DMFS model for the first time to reconstruct Holocene January and July mean temperature sequences. Results showed regional consistency in temperature variability. Xiadawu and Zhongda profiles fossil pollen assemblages and reconstructed temperatures indicated: 12.5–6.0 ka BP climatic amelioration occurred; 6.0–4.0 ka BP a warm-humid phase was confirmed(HTM); 4.0–2.5 ka BP the climate became cold and dry; 2.5–0.5 ka BP temperatures began to rise.The reconstruction aligned with multiple high-resolution environmental records across the QTP in both trend and dry-wet event timing (despite minor chronological offsets), thereby confirming its reliability.

In the analysis of proxy indicators, the focus should be on the experimentation and collection of high-resolution multiple proxies. In terms of climate reconstruction, it is essential to leverage the strengths of multiple disciplines to provide more precise methods for future climate reconstructions. For instance, the application of methods. Machine learning algorithms, when applied to climate reconstruction, can deepen our understanding of past climate dynamics and thereby enable corresponding predictions of future climate change.
